# Prioritizing peptides for targeted mass spectrometry experiments using deep learning

**DOI:** 10.64898/2026.05.21.727053

**Published:** 2026-05-26

**Authors:** Shreyash Sonthalia, Priank Dasgupta, Chris Hsu, Bo Wen, Michael J. MacCoss, William Stafford Noble

**Affiliations:** 1 Department of Genome Sciences, University of Washington; 2 Paul G. Allen School of Computer Science and Engineering, University of Washington

## Abstract

One critical step in any targeted mass spectrometry experiment is selecting, from each protein of interest, a small number of peptides that respond well in the mass spectrometer and can serve as reliable proxies for protein quantification. Existing methods select target peptides either by relying on prior empirical measurements, limiting their applicability to previously observed peptides, or using machine learning to predict peptide behavior from sequence alone. However, current machine learning tools suffer from various limitations, including using detectability as an indirect proxy for intensity, relying on small training sets, or ignoring the precursor charge state. In this study, we introduce Bromo, a transformer-based deep learning model that ranks peptide precursors from a given protein by their relative response, taking charge state into account. Trained on millions of annotated peptide pairs derived from large-scale, publicly available data-independent acquisition mass spectrometry data, Bromo consistently outperforms existing sequence-based methods across diverse, independent datasets. Furthermore, we show that finetuning Bromo on experiment-specific data can account for differences in sample preparation, sample matrix, and instrument platform, all of which influence which peptides serve as optimal targets. This adaptability makes Bromo a practical tool for selecting target peptides for selected reaction monitoring and parallel reaction monitoring assay development across a wide range of experimental conditions.

## 1 Introduction

Targeted mass spectrometry (MS) coupled with liquid chromatography (LC) enables sensitive and reproducible quantification of proteins in complex biological samples by monitoring a selected set of target peptides using approaches such as selected reaction monitoring (SRM) [1–3] or parallel reaction monitoring (PRM) [4, 5]. Targeted mass spectrometry is widely used for applications such as biomarker detection and verification of results from untargeted data-dependent acquisition (DDA) and data-independent acquisition (DIA) analysis [6, 7]. One critical step in designing a targeted assay is selecting, from each protein of interest, a small number of peptides to monitor for the protein of interest. The quality of these peptide selections directly determines assay performance: ideal targets should yield strong, reproducible signals in the mass spectrometer and serve as reliable proxies for protein abundance.

A peptide’s response in the mass spectrometer depends on its amino acid sequence, the sample matrix, the instrument platform, and the acquisition method, making peptide selection a challenging problem. Early targeted assay development relied on DDA experiments to identify candidate peptides and to generate library spectra from which SRM transitions (i.e., pairs of corresponding precursor and product ions) could be derived [8]. Although DDA’s stochastic sampling makes it unreliable as a direct predictor of peptide response in a targeted assay [9], the resulting library spectra provided a practical starting point for transition selection. To address the shortcomings of DDA-based selection, the field moved toward empirical refinement of assays directly in the target sample matrix using SRM or PRM [10], iteratively evaluating candidate peptides on the target instrument. However, this strategy becomes difficult when the target protein is near the instrument’s limit of detection, where it is often unclear whether the observed signal corresponds to the intended peptide of interest. To avoid this ambiguity, an alternative empirical strategy is to produce candidate proteins recombinantly via in vitro transcription and translation and then directly measure their tryptic peptides in isolation [9, 11]; this approach yields high-quality peptide selections but is time consuming and expensive. More recently, several groups have demonstrated that DIA data can efficiently inform peptide selection by providing comprehensive measurements of peptide behavior under conditions closely related to targeted acquisition, using DIA chromatogram libraries to select and schedule peptide targets for SRM and PRM assays [7, 12–14]. Although empirical approaches work well when relevant measurements are available, they cannot generalize to proteins that have not previously been observed and may not perform well in new experimental conditions.

An alternative strategy is to use machine learning to predict a peptide’s response directly from its amino acid sequence, removing the dependence on prior measurements. The earliest work in this direction used DDA data to predict which peptides will be reliably detected [15, 16]; however, as noted above, DDA-based peptide selection is suboptimal for targeted proteomics experiments. More recent tools, such as PeptideRanger [17], use random forest models trained on large, diverse collections of mass spectrometry experiments but still rely primarily on DDA data and predict detectability rather than quantitative intensity. Another related method, d::pPop, uses hand-crafted physicochemical peptide features together with a five-layer, fully connected neural network trained on DDA data to predict peptide observability scores and induce a within-protein ranking [18]. One notable exception to the reliance on DDA is PREGO [19], which trains a neural network on features derived from a set of equimolar synthetic peptides measured by DIA; however, the small, synthetic training set limits the diversity of peptide behavior the model can capture. In general, existing sequence-based tools suffer from various limitations, including using detectability as an indirect proxy for response, relying on small training sets that may not capture the diversity of peptide behavior in biological samples, or ignoring the precursor charge state.

The approach we describe here addresses these limitations by framing peptide selection as a relative ranking task. To select peptides for a given protein, it is sufficient to rank the candidate peptides relative to one another rather than predict their absolute responses. Our deep learning model, Bromo, takes as input pairs of peptide precursors from the same protein and predicts which will yield a larger quantitative response in a DIA experiment. This pairwise ranking model can then be used to induce a full ranking of peptides for a given protein. Bromo uses a transformer architecture [20] and was trained on millions of peptide pairs derived from large-scale, publicly available DIA data. We evaluate Bromo against existing sequence-based methods across diverse, independent DIA datasets, and assess whether fine-tuning can adapt the model to new experimental conditions.

## 2 Results

### 2.1 The Bromo model learns to rank peptide pairs

**Figure 1:**
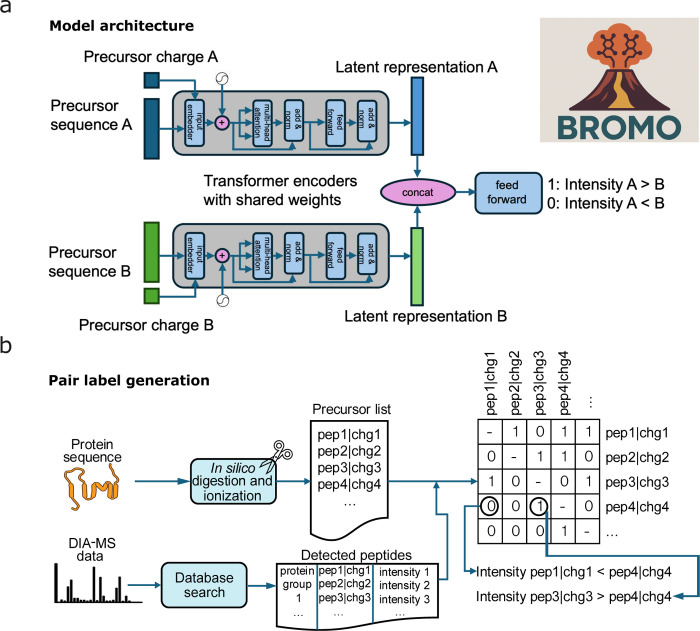
Overview of peptide ranking. (a) Schematic overview of the Bromo model architecture. (b) Training data generation for peptide ranking. The binary label at position (i,j) in the label matrix indicates whether precursor i exhibits higher intensity than precursor j.

To effectively prioritize peptides within a protein for detection in a targeted proteomics experiment, we built a deep learning model called Bromo that can be used to rank peptide precursors (i.e., peptide sequences with specific charge states) within a protein based on their predicted MS2 response signals in DIA experiments. As shown in Figure 1a, Bromo takes as input an ordered pair of precursors from the same protein and predicts whether the first precursor will yield higher intensity than the second precursor in a DIA experiment. The underlying assumption is that peptides from the same protein are expected to have the same initial concentration in the sample, and the difference in their response signals in a DIA experiment is mainly due to the sequence features of the peptides and the variation introduced during sample preparation (e.g., protein digestion) and peptide ionization. In general, for a set of peptides derived from the same protein, selecting peptides with higher intensities in a DIA experiment is preferred for targeted proteomics experiments [12, 13, 19]. We hypothesize that a model trained directly on peptide response signals can rank peptides for targeted proteomics experiments more effectively than models that predict simpler observability metrics such as peptide detectability [16, 17]. In addition, our approach does not rely on synthetic datasets for model training; instead, it can be trained directly on DIA datasets from complex samples (Figure 1b). Bromo can also be fine-tuned on data generated from different MS instruments to improve performance under specific experimental settings.

### 2.2 Bromo outperforms existing methods for peptide ranking

**Figure 2:**
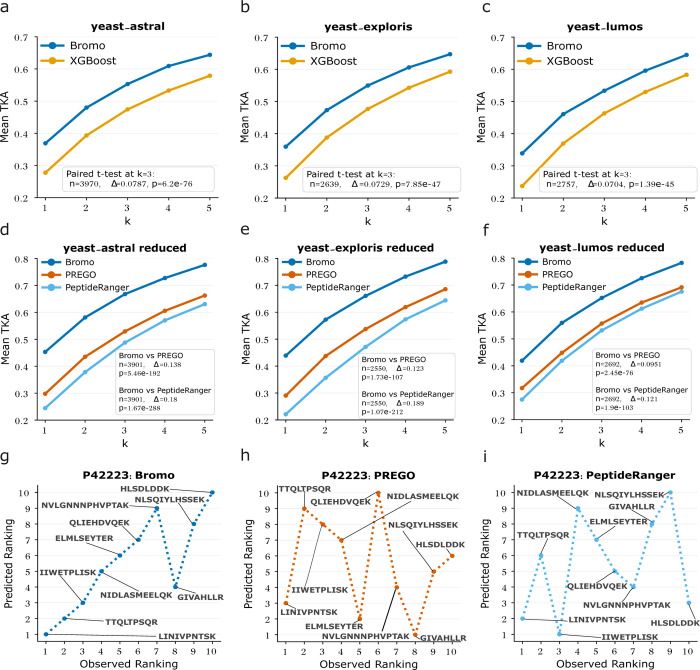
Peptide ranking performance on three DIA datasets. The figure plots the top-k accuracy (TKA) for k=[1,5] on paired yeast datasets from three different MS instruments (Astral: **(a)** and **(d)**, Exploris: **(b)** and **(e)** and Lumos: **(c)** and **(f)**). In panels **(a)–(c)**, the blue curve is the TKA using Bromo, and the orange curve is the TKA using the baseline XGBoost model. For panels **(d)–(f)**, the blue curve is the TKA using Bromo, the red curve is the TKA using PREGO, and the light blue curve is the TKA using PeptideRanger. **(g)–(i)** Rankings of peptides from protein P42223 using Bromo, PREGO, and PeptideRanger, respectively.

To evaluate the performance of our approach, we benchmarked Bromo across three independent datasets acquired on three different MS platforms (Orbitrap Astral, Orbitrap Fusion Lumos, and Orbitrap Exploris 480), as shown in Table 1. For each dataset, we trained the model on proteins detected in a human DIA dataset and tested its performance on a paired yeast DIA dataset generated under identical LC-MS/MS experimental conditions. This evaluation setup ensures that high performance on the test set reflects genuine generalization rather than memorization of training peptides. We first compared Bromo against a baseline method that uses a standard machine learning classifier, XGBoost [21] (see Methods for details), trained and evaluated using the same data splits. Bromo consistently outperformed the XGBoost baseline, achieving significantly higher top-k accuracy (TKA) across all three evaluation sets (Figure 2a–c).

We next benchmarked Bromo against PREGO and PeptideRanger across the same three evaluation sets. Because these tools do not natively model precursor charge states, we derived peptide-level rankings from Bromo’s precursor-level predictions for a fair comparison (see Methods). Comparisons were further restricted to peptides present in the rankings output by each tool. On this reduced yeast-astral dataset, Bromo yielded significantly higher TKA values at k=3 than both PREGO (paired t-test, *p* = 5.46 × 10^−192^) and PeptideRanger (paired t-test, *p* = 1.67×10^−288^) (Figure 2d–f). An illustrative example of peptide ranking by the three methods is shown in Figure 2g–i, in which a randomly sampled protein group from the yeast-astral dataset was sampled and peptide-level rankings were computed.

Notably, while PREGO was explicitly designed to model peptide ranking, PeptideRanger was trained to predict general peptide observability (i.e., whether a peptide is detected across a broad array of mass spectrometry experiments) and uses this detectability as a proxy for ranking. This fundamental difference in training objectives likely accounts for PeptideRanger’s relatively poor performance on this targeted selection task.

### 2.3 Instrument-specific fine-tuning mitigates cross-platform variability

Mass spectrometry instrument characteristics can profoundly influence peptide detectability and relative response. To quantify this variability and evaluate strategies to overcome it, we assessed the consistency of peptide rankings across different MS platforms and tested whether instrument-specific fine-tuning improves Bromo’s predictive performance.

**Figure 3:**
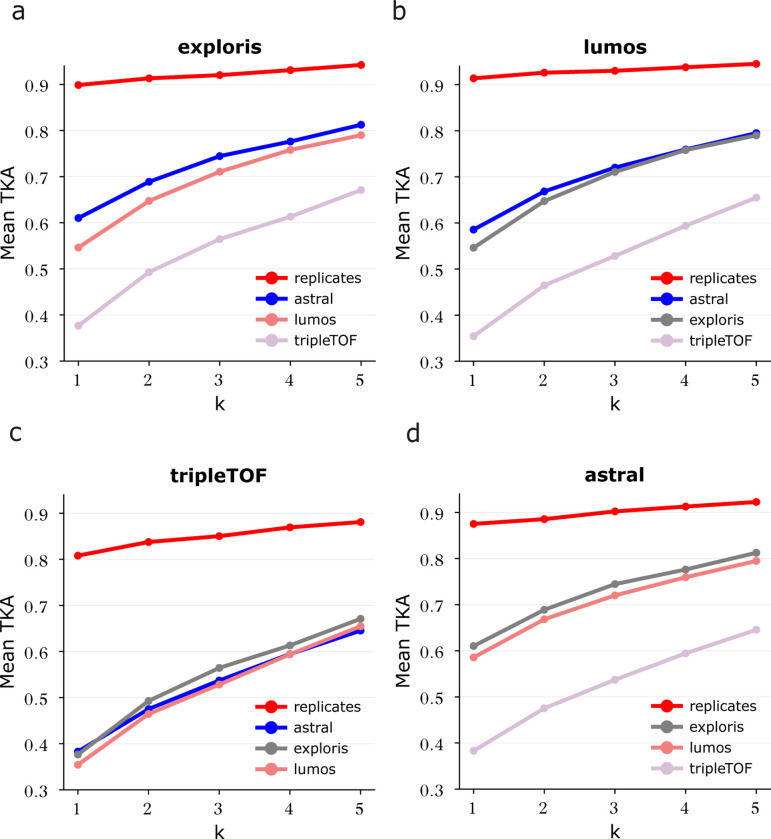
Peptide response varies across different instruments. **(a)–(d)** Peptide ranking consistency across replicates and instruments. All data used in these analyses were from human cell line samples. For intra-instrument comparisons, shown as red lines, one replicate from each dataset was taken as the gold standard and the other replicate from the same dataset was taken as the test ranking. For cross-instrument comparisons, the instrument platform listed at the top of each plot is used as the gold standard, and TKA is calculated with respect to replicates from three different platforms, listed in the legend of each plot.

We first established baselines for intra-instrument and inter-instrument consistency of peptide rankings. To do this, we began by comparing rankings derived from technical replicate measurements on a single instrument platform. We arbitrarily selected one replicate as the gold standard and computed TKA for the ranking from the other replicate relative to that gold standard. As shown in Figure 3a–d, TKA values at k=3 on the four datasets, each derived from a distinct MS platform, ranged between 0.812 and 0.918. This result confirms that peptide ranking remains highly consistent across independent runs acquired with identical instrument settings. Next, we repeated this experiment using replicates acquired on different types of instruments. Again, we selected one instrument platform as the gold standard and computed TKA based on the ranking from the other instrument platform. This inter-instrument analysis revealed a substantial reduction in ranking consistency, measured using TKA at k=3, to a range of 0.528 to 0.745 (Figure 3a–d), highlighting the considerable variability in rankings produced by different mass spectrometer platforms.

**Figure 4:**
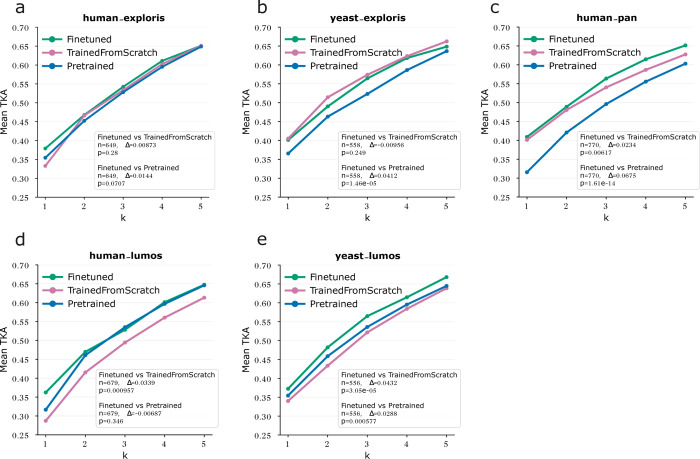
Evaluating model fine-tuning for peptide ranking on different datasets. Three types of models (fine-tuned models, models trained from scratch, and a pretrained model) were compared on five datasets: **(a)** human-exploris, **(b)** yeast-exploris, **(c)** human-pan, **(d)** human-lumos, and **(e)** yeast-lumos.

We next investigated whether fine-tuning could adapt a pretrained Bromo model to a different type of instrument. To this end, we compared the performance of a Bromo model trained using the human-astral dataset with the performance of the same model after it had been fine-tuned using data from the same instrument as the test data (Exploris, Lumos, or SCIEX TripleTOF). We observed that, in each case, performance after fine-tuning was either better than or not significantly different from that of the pretrained model, though the degree of improvement varied (Figure 4). For the human-pan dataset, generated on a SCIEX TripleTOF, the TKA at k=3 improved by 0.0675 (from 0.497 to 0.565). In contrast, the change in TKA at k=3 for the human datasets from the Exploris and Lumos instruments was not significant and relatively small (0.0144 and −0.00069, respectively).

We then compared fine-tuned models with models trained from scratch on each dataset. As shown in Figure 4, fine-tuning yielded better or comparable performance on most datasets relative to models trained from scratch. Notably, while the human-pan dataset benefited significantly from fine-tuning, the Exploris and Lumos human datasets did not exhibit similar improvements. Fine-tuning, however, was never significantly worse than the pretrained model or the model trained from scratch on each dataset. This result suggests that while fine-tuning is a viable strategy for mitigating cross-platform variability in some contexts, its overall efficacy depends on the specific instrument and dataset characteristics.

### 2.4 Bromo’s performance scales with increasing training data

**Figure 5:**
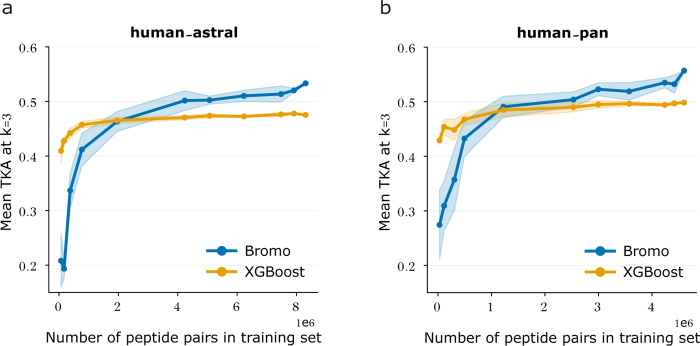
Evaluating the impact of training data size on model performance. Performance was evaluated on two datasets: **(a)** the human-astral dataset and **(b)** the human-pan dataset. On each dataset, two models were evaluated: a Bromo model and an XGBoost model. The x-axis shows the number of peptide pairs in each training set while the y-axis shows the mean TKA at k=3 on the validation set. Shaded regions correspond to the standard deviation across 5 random subsamples of the training set.

To evaluate the impact of training set size on performance, we plotted ranking accuracy as a function of the number of protein groups used for training. This analysis, performed on both the human-astral and human-pan datasets, allowed us to compare how well each model scales with additional data. As illustrated in Figure 5, Bromo’s performance exhibits steady improvement as the number of training peptide pairs increases. In contrast, the performance of the XGBoost baseline model quickly plateaus. This divergence highlights the capacity of Bromo’s deep learning architecture to effectively leverage increasing amounts of training data for superior peptide ranking, an advantage that traditional machine learning methods fail to capture.

### 2.5 Comparing binomial and majority voting labeling schemes

**Figure 6:**
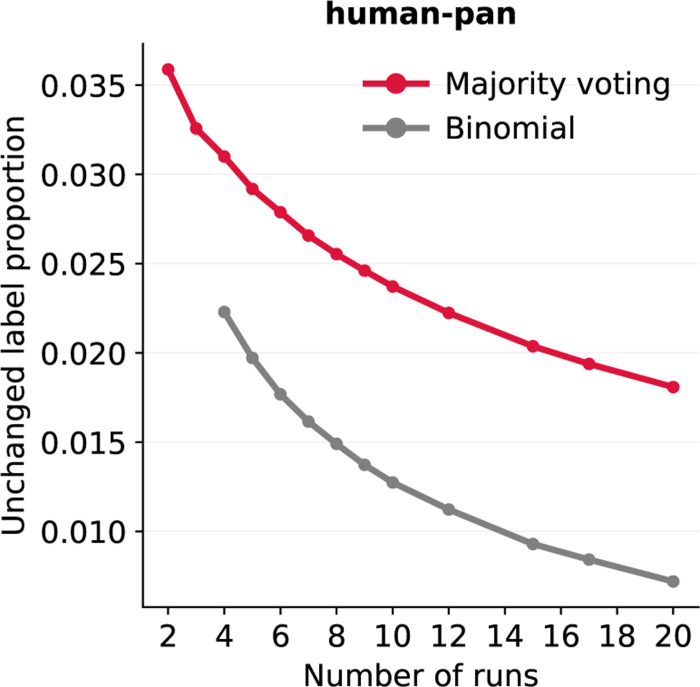
Comparing consistency of labeling schemes. The figure plots the proportion of peptide pairs that receive different labels using the full human-pan dataset versus a downsampled version of the same data. The x-axis is the number of runs in the downsampled data, and the two series correspond to label assignment using the binomial distribution versus majority voting. With fewer than 5 runs, the binomial labeling scheme assigns all zero labels; thus, only the majority voting curve has values for fewer than 4 runs.

In general, a desirable property of a labeling scheme is that, for a given pair of peptides, the assigned label should be unlikely to change as the number of observations increases. To quantify the consistency of each of our two labeling schemes with respect to the number of observations, we carried out a downsampling simulation. Using the human-pan dataset, we assigned each peptide pair a “full data” label based on all 6864 available runs, using either the binomial assignment or the majority voting procedure depending on the number of runs in which the peptide pair was detected. We then randomly selected 20 runs, re-computed the labels, and counted how many peptide pairs received a “downsampled” label that differed from the “full data” label; we repeated this procedure while iteratively discarding one additional run at each step. The resulting curves (Figure 6) suggest that while the binomial distribution labeling scheme yields labels that are more consistent, the majority voting scheme does not perform significantly worse: for both schemes, over 99% of peptide pairs detected in at least 5 runs received the same label across all 20 runs.

## 3 Discussion

Bromo addresses a central problem in targeted proteomics assay development: selecting, from the candidate peptides for a protein of interest, those most likely to produce strong and reliable mass spectrometry signals. Rather than predicting peptide response signals on an absolute scale, Bromo directly models the relative response of peptide precursors (i.e., peptide sequences with specific charge states) from the same protein. This formulation is well matched to the practical decision made during assay design, where the goal is usually not to estimate an absolute peptide intensity but to choose a small number of high-responding peptides. Across independent DIA datasets generated on multiple instrument platforms, this pairwise ranking approach outperformed both a conventional XGBoost baseline trained on the same data and the existing sequence-based tools PREGO [19] and PeptideRanger [17]. These results suggest that DIA datasets with deep peptide and protein coverage provide a useful empirical basis for learning peptide response properties relevant to targeted assay development.

Three design choices drive this performance. First, Bromo uses observed precursor intensity, rather than peptide detectability, as the training signal. Detectability is useful for identifying peptides that are likely to be observed, but it does not distinguish among the subset of detectable peptides that differ substantially in quantitative response; this distinction is reflected in Bromo’s large advantage over PeptideRanger. Second, Bromo models peptide precursors, including charge state, rather than peptide sequences alone, because different charge states of the same peptide can have different ionization efficiencies, fragmentation behaviors, and suitabilities for targeted acquisition. Third, the pairwise framing allows the model to learn from within-protein comparisons in complex biological samples, where peptides from the same protein share the protein’s abundance, so observed intensity differences are attributable to peptide-specific response. A pairwise objective is also better matched to the assay-design decision than pointwise intensity regression: the user only needs to know which peptides are best within a given protein, not the absolute intensity any peptide will produce. This framing, combined with the use of complex biological samples, sidesteps the need for equimolar synthetic peptide datasets like those used to train PREGO [19].

Beyond these design choices, the training-size analysis indicates that Bromo can take advantage of additional data, whereas the XGBoost baseline quickly plateaus, suggesting that performance may continue to improve as larger and more diverse DIA resources become available. This scaling behavior also argues for prioritizing the curation of public DIA corpora as the most direct path to better peptide-ranking models.

The cross-instrument analyses emphasize that peptide response is not an intrinsic property of sequence alone. Replicate measurements acquired under the same instrument conditions yielded highly consistent peptide rankings, whereas rankings were substantially less consistent across different instrument platforms. This observation helps explain why a single universal peptide-ranking model is unlikely to be optimal for all experimental settings. Fine-tuning provides a practical way to adapt a pretrained model to new datasets, sample matrices, and instruments. In our experiments, fine-tuned models performed comparably to or better than models trained from scratch on most datasets, though the magnitude of improvement varied. This variation likely reflects two factors: the divergence between target and pretraining conditions, and the amount of representative data available for adaptation. In practice, Bromo should be viewed as a strong starting point for assay development; we recommend fine-tuning over training from scratch whenever DIA data from the target instrument are available, even at modest scale.

As with any supervised learning model, Bromo’s accuracy is limited by the size, quality, and diversity of its training data. The current model was trained and evaluated primarily using tryptic peptides and DIA-derived MS2 precursor-level response signals, so its performance may be reduced for other proteases, unusual peptide modifications, uncommon charge states, depletion or enrichment workflows, or sample matrices that are poorly represented in the training data. The model also inherits limitations from how labels are derived from DIA-NN output: missing peptide detections, interference, protein inference ambiguity, and intensity normalization can all affect the observed ranking used as ground truth. Even with high-quality data, replicate measurements on a single instrument yielded TKA values at k=3 between 0.812 and 0.918, suggesting that the gold-standard ranking is itself noisy and that achievable model performance is bounded above by this replicate ceiling rather than by 1.0. Our comparison of binomial and majority-voting labeling schemes suggests that consensus labels are generally stable when multiple runs are available, but low-replicate datasets provide noisier supervision; this argues for prioritizing replicate depth over breadth when assembling future training corpora. More generally, future training sets that include a wider range of sample types, more instrument platforms, and carefully curated replicate measurements should improve both model accuracy and the reliability of fine-tuning.

Bromo solves only one part of targeted assay design. Selecting high-responding peptides is necessary, but a complete SRM or PRM method also requires choosing transitions or fragment ions, predicting or measuring retention times, avoiding interferences, and scheduling targets within instrument duty-cycle constraints. Practical deployment of Bromo should therefore combine its peptide-ranking output with tools for retention time prediction or calibration [22–26], fragment ion intensity prediction [24–27], transition selection, and scheduling optimization. Even with this integration, empirical validation remains important for low-abundance proteins, clinically oriented assays, and experiments in complex matrices where interference can dominate the observed signal. Together, these components would allow Bromo to prioritize candidate peptides while downstream assay-design software determines which precursor and fragment ions can be measured reliably in a specific method.

Overall, these results support the use of DIA data with deep proteome coverage and pairwise deep learning for peptide selection in targeted proteomics. Bromo improves sequence-based peptide ranking by aligning the learning objective with the assay-design decision, incorporating precursor charge state, and allowing adaptation to experiment-specific conditions. As public DIA datasets continue to grow and as peptide ranking is integrated with complementary prediction [25, 26] and scheduling tools, Bromo should reduce the empirical burden of targeted assay development while preserving the flexibility needed for diverse biological and analytical applications.

## 4 Methods

### 4.1 The Bromo deep learning model

Bromo is a deep learning model that takes as input pairs of peptide precursors (peptide sequences and associated charge states) from the same protein and predicts whether the first precursor will yield a larger quantitative response than the second precursor in a DIA mass spectrometry experiment. The model consists of twin transformer encoders, sharing the same architecture and parameters, followed by fully-connected layers for binary classification. The encoder input is a list of integers, where each integer represents the token for an amino acid. This input is extended with a pad token to a maximum peptide length of 30. The encoder consists of four layers: an embedding layer with output dimension 128, a positional encoder layer for each token, and four transformer layers, each with 8 attention heads and a feedforward dimension of 512. The output token representations are aggregated into a single 128-dimensional vector via learned attention pooling, where a learned scoring function assigns a weight to each token position and the final representation is the weighted sum. The vector representations of the two peptides are concatenated together with the two charge state embeddings (dimension 8), yielding a 272-dimensional joint representation, which is fed into two fully connected layers with dimensions 256 and 2, respectively. Dropout was applied to the transformer layers and the fully connected classifier layers. The model outputs logits over two classes, corresponding to which of the two peptide-charge combinations is predicted to yield a larger MS2 intensity value. The model optimizes the cross-entropy loss function using the Adam optimizer [28]. After training, the best-performing model was selected using the area under the ROC curve, calculated on the validation set.

### 4.2 Deriving precursor-level and peptide-level rankings from precursor-pair binary classification

The goal of Bromo is to induce a ranking over all the peptides (in each possible charge state) in the protein. To do this, binary classification results for each precursor pair within a protein are used to derive a precursor-level ranking. We induce a ranking by running inference for each peptide+charge pair combination in the protein and sorting by the number of times they are assigned a positive label by the model, with ties broken by the average predicted score assigned to the peptide. For each pair, inference is run for both directions (i.e., (A,B) and (B,A)) and an equally weighted convex combination of the two predictions is used as the representative score for the pair. The tie-breaking step is necessary because the model does not always obey transitivity.

For comparison to existing methods that do not consider charge state [17, 19], we derive a peptide-level ranking by choosing a representative precursor for use in peptide-level ranking. We do this by taking the precursor-level ranking, keeping only the precursor with the highest average predicted score as the representative peptides, and then sorting by the average predicted score assigned to the peptide to break ties.

### 4.3 datasets

A total of seven datasets were used in the study (Table 1). The first dataset, human-astral, was used for pretraining because this dataset contained the largest number of detected peptides. We used the other six datasets for test-sets and/or finetuning. The yeast datasets were also from the same study as human-astral and were used for finetuning and testing the base Bromo model because they contained protein groups and peptides not found in the human datasets (ProteomeXchange: PXD056793) [25]. The human-pan dataset was generated from 949 cancer cell lines across 28 tissue types analyzed by SCIEX 6600 TripleTOF mass spectrometers. This dataset includes a total of 6864 DIA runs, and was also used for fine-tuning the model because this dataset contained the largest number of independent DIA runs, and thus precursor-pair labels with lowest variance. DIA-NN peptide detection results were downloaded from PRIDE with identifier PXD030304.

The two other human datasets were generated from different instruments and were also from the same study as human-astral (ProteomeXchange: PXD056793) [25]. These datasets were also used for fine-tuning. Each dataset was analyzed using DIA-NN (version 1.8.1) [29], and the normalized precursor intensity (“Precursor.Normalised”) from DIA-NN’s main report was used as the precursor intensity.

### 4.4 Assigning labels to peptide pairs

For a given set of one or more MS runs, a collection of labeled precursor pairs was created in three steps. For each individual MS run and protein group, we first generate all possible precursor pairs, excluding self comparisons and excluding any precursor that maps to multiple proteins. This is done by performing *in silico* tryptic digestion of each protein sequence using the Pyteomics package (https://github.com/levitsky/pyteomics), allowing one missed cleavage, peptide length 7–30, and assigning charge states of 2, 3, and 4 to each peptide. Second, for each pair of peptide-charges (A,B), if both A and B were detected in the run, then a label is assigned based on whether A has a higher observed intensity (label “1”) or a lower observed intensity (label “0”). If A was detected but not B, then the label “1” is assigned, and vice versa if B was detected but not A. If neither A nor B was detected in the run, then the pair is discarded. As a form of data augmentation to induce equivariance to the order of the input peptides, each pair is included in the dataset twice (i.e., (A,B) and (B,A)), with opposite labels.

Finally, a consensus label is assigned to each pair across all the runs in the dataset. Say that, after the two-step procedure outlined above, a given pair (A,B) has a label assigned for n runs, and that the label is positive in m of those runs. For large datasets (human-pan in Table 1), we use the binomial distribution to calculate the probability of observing at least m heads in n flips of a fair coin:

$$P(X\leq m)=\sum_{k=0}^{m}\left(\begin{array}{c} n\\ k \end{array}\right)\frac{1}{2^{n}}$$

We set the consensus label to “1” if 1−P(X≤m−1)<τ and “0” if P(X≤m)<τ. In this work, we use τ=0.05. The binomial distribution method was only used for peptide pairs that were detected in at least five runs, with a simple majority voting scheme used for the remaining peptide pairs. For the remaining, smaller datasets, we assign the consensus label using the majority voting scheme.

### 4.5 Splitting the data for training, validation, and testing

For the human-astral dataset, we randomly split the protein groups into training, validation, and testing sets with a ratio of 0.6, 0.2, and 0.2, respectively. The training set was used to train the model, the validation set was used to decide upon training convergence, and the testing set was used to evaluate the model performance.

For other human datasets used for finetuning, we created train/validation/test splits that respect the original splits created for the human-astral dataset. This is done by identifying any peptide pairs that are shared between the new dataset and the human-astral training set. Any protein group in the new dataset that contains at least one such shared peptide pair is added to the new training set. We then split the remaining protein groups randomly, to achieve overall train/validation/test ratios of 0.6, 0.2, and 0.2. For the yeast datasets used for finetuning, we created train/validation/test splits on protein groups with a ratio of 0.6, 0.2, and 0.2, respectively.

### 4.6 Baseline methods

We compared the Bromo model to three baseline methods: PREGO, PeptideRanger and XGBoost.

PREGO was downloaded from https://github.com/briansearle/intensity_predictor. We used PREGO’s classify command to generate a score for each peptide. For all pairs of peptides from a single protein, the probability of peptide $p_{1}$ having a higher intensity than peptide $p_{2}$ was then calculated as follows:

(1) $$p\left(p_{1},p_{2}\right)=\frac{s\left(p_{1}\right)}{s\left(p_{1}\right)+s\left(p_{2}\right)}$$

where s(p) is the score assigned by PREGO to peptide p.

PeptideRanger was downloaded from https://github.com/rr-2/PeptideRanger. The function create_peptidome was first used to generate a list of peptides from each protein, using the following parameters: missed_cleavages = c(0,1), synth_peps = FALSE and aa_range = c(7,35). Then the function prioritize_peptides was used to predict the detectability of each peptide with the following parameters: max_n = 100, prediction_model = RFmodel_ProteomicsDB, priorities = “RF_score” and priority_thresholds = 0. The detectability score (RF_score) was then used to rank pairs of peptides from the same protein, and these scores were converted to pairwise scores using Equation 1.

In addition, we trained an XGBoost classifier on inputs representing the amino acid composition and charge state of each peptide. Specifically, each peptide is represented as a length-22 vector containing the charge state as well as integer counts of each possible amino acid. Each canonical amino acid was represented by a separate dimension in the vector, whereas non-canonical amino acids were represented by a single dimension. These two vectors are concatenated and provided as input to XGBoost. We trained the classifier using the same training sets that we used to train Bromo.

### 4.7 Performance measures

To evaluate the performance of the various models, we designed a performance measure that quantifies the quality of a given set of selected peptides. Based on the ranking of peptides produced by a given method, we select a specified number k of top-ranked peptides to target, discarding any protein group with k or fewer unique peptides. We then compare the top-k predicted peptides to the gold standard ranking of peptides by their observed intensities. The performance measure, the top-k accuracy (TKA), is the fraction of predicted peptides that are included in the top k according to the gold standard:

$$\mathrm{TKA}\left(q_{1},\ldots ,q_{k},p_{1},\ldots ,p_{k}\right)=\frac{1}{k}\sum_{i=1}^{k}\sum_{j=1}^{k}\delta \left(q_{i},p_{j}\right)$$

where $q_{1},\ldots ,q_{k}$ are the top k predicted peptides, $p_{1},\ldots ,p_{k}$ are the top k peptides in the gold standard, and δ(q,p) is 1 if q=p and 0 otherwise. The value of TKA ranges between 0 and 1, with larger values being better. In practice, we compute average TKA values across all proteins in a given dataset, and we repeat the calculation for varying values of k. To assess the statistical significance of a given observed difference in performance between two methods, we use a paired t-test on the per-protein differences in TKA.

### 4.8 Peptide ranking comparison across experimental replicates and instruments

To evaluate precursor ranking consistency across experimental replicates and instrument platforms, we compared precursor rankings derived from pairs of DIA runs acquired on Orbitrap Astral, Orbitrap Fusion Lumos, Exploris 480, and SCIEX 6600 TripleTOF instruments. For each comparison, we restricted the analysis to proteins for which the same two peptides were detected in both runs and generated all within-protein precursor pairs. One run was treated as the observed ranking, with pairwise labels assigned according to which precursor had the larger DIA-NN normalized precursor intensity, and the other run was treated as the predicted ranking, with pairwise scores defined by the corresponding precursor intensity ratios. For comparisons across experimental replicates, the two runs were drawn from the same dataset. For cross-instrument comparisons, the observed ranking was derived from DIA data acquired on one instrument and the predicted ranking was derived from DIA data acquired on a different instrument. We then quantified agreement between the observed and predicted rankings using the same pairwise ranking analysis used throughout the manuscript.

## Figures and Tables

**Table 1:** Summary of datasets used in the study. A peptide is counted in the “Peptides” column if it participates in at least one peptide pair.

dataset	Instrument	Sample	Runs	Protein groups	Peptides	Peptide pairs

human-astral	Orbitrap Astral	HeLa cells	4	8070	443778	18384919
human-pan	6600 TripleTOF	Cancer cell lines	6864	6777	280765	10504865
yeast-astral	Orbitrap Astral	Yeast	4	4710	317958	12777431
human-lumos	Orbitrap Fusion Lumos	HeLa cells	3	5879	230799	6892272
yeast-lumos	Orbitrap Fusion Lumos	Yeast	3	4100	187053	5346828
human-exploris	Exploris 480	HeLa cells	4	5521	220125	6964014
yeast-exploris	Exploris 480	Yeast	4	3971	185481	5679852

## Data Availability

The paired human and yeast DIA MS/MS data with accession number PXD056793 [25] were downloaded from https://panoramaweb.org/Carafe.url. The large human cell line data was downloaded from PRIDE with accession number PXD030304. All search results, intermediate and processed data as well as model checkpoints used for reproducing the results in this paper are available through Zenodo with the link provided in the bromo-manuscript repository (https://github.com/Noble-Lab/bromo-manuscript).

## 4.10 Code availability

Bromo is open source and is available with an Apache 2.0 license at https://github.com/Noble-Lab/bromo. The scripts used to perform the analyses in this paper and reproduce figures and results are available at https://github.com/Noble-Lab/bromo-manuscript.
